# Comprehensive Dental Management Under General Anesthesia in a Pediatric Patient With Type 3 von Willebrand Disease: A Case Report

**DOI:** 10.1155/crid/4211452

**Published:** 2026-09-07

**Authors:** Samira Sadigh Batha, Nazanin Nasr, Nasser Kaviani

**Affiliations:** ^1^ Dental Students′ Research Committee, Department of Pediatric Dentistry, School of Dentistry, Isfahan University of Medical Sciences, Isfahan, Iran, mui.ac.ir; ^2^ Department of Oral and Maxillofacial Surgery, Dental Research Center, Dental Research Institute, School of Dentistry, Isfahan University of Medical Sciences, Isfahan, Iran, mui.ac.ir

**Keywords:** bleeding disorder, dental care, general anesthesia, pediatric dentistry, Type 3, von Willebrand disease

## Abstract

**Background:**

Type 3 von Willebrand disease (VWD) is the most severe and rare form of this inherited bleeding disorder, characterized by near‐complete absence of von Willebrand factor (VWF) and markedly reduced Factor VIII activity. These patients are at high risk of excessive bleeding during invasive procedures, including dental treatment.

**Case Presentation:**

A 4‐year‐old boy with a confirmed diagnosis of Type 3 VWD presented with intermittent pain in the maxillary left lateral incisor, without clinical or radiographic signs of an abscess. Comprehensive dental rehabilitation was performed under general anesthesia. Treatment consisted of pulpotomy and placement of stainless‐steel crowns for primary molars, as well as pulpectomy and resin composite restorations for primary incisors. Hemostatic management included preoperative and postoperative pdVWF/F VIII concentrate administration in consultation with a hematologist.

**Conclusion:**

With meticulous multidisciplinary planning, appropriate perioperative factor replacement, and careful intra‐ and postoperative management, safe and effective dental rehabilitation can be achieved in pediatric patients with Type 3 VWD.

## 1. Introduction

Von Willebrand disease (VWD) is the most common inherited bleeding disorder worldwide and results from quantitative or qualitative defects in von Willebrand factor (VWF), a critical glycoprotein involved in primary hemostasis and carrier of coagulation Factor VIII (FVIII) in plasma. VWD accounts for approximately 26% of all hemostasis disorders and affects males and females equally [1]. According to the current classification, VWD is categorized into three major types: Type 1, characterized by partial quantitative deficiency of VWF; Type 2, involving qualitative defects with several subtypes; and Type 3, which is marked by a near‐complete absence of VWF and very low FVIII levels. Type 1 VWD accounts for up to 70% of cases, whereas Types 2 and 3 represent approximately 20% and 5% of patients, respectively [2]. Type 3 VWD is rare and associated with the most severe bleeding phenotype, including mucocutaneous bleeding, joint and muscle hemorrhages, and a significantly increased risk of perioperative bleeding during surgical procedures such as dental treatments or tooth extractions [3, 4]. Patients with Type 3 VWD typically lack a sufficient response to desmopressin (DDAVP) due to the absent VWF stores, necessitating replacement therapy with plasma‐derived or recombinant VWF/FVIII concentrates for achieving adequate hemostasis.

The dental management of patients with bleeding disorders, including VWD, depends on both the severity of the underlying condition and the extent of the planned dental intervention. Minimally invasive procedures are performed in patients with mild bleeding tendencies, often requiring little to no modification of routine treatment protocols. In contrast, patients with severe bleeding disorders necessitate meticulous planning aimed at optimizing hemostasis and maintaining bleeding control through appropriate local and adjunctive measures [5].

Recent clinical guidelines emphasize the importance of individualized hemostatic protocols considering VWD type, history, and risk of bleeding, and risk of thrombosis. This may include perioperative administration of factor concentrates, antifibrinolytic agents (e.g., tranexamic acid), and close multidisciplinary collaboration between dental practitioners and hematologists to minimize hemorrhagic complications [6, 7]. Dental treatment poses unique challenges in patients with bleeding disorders due to the highly vascular nature of the oral cavity, where even minimally invasive procedures may trigger prolonged bleeding. Evidence indicates that thorough preoperative assessment and carefully coordinated perioperative management substantially reduce the risk of intra‐ and postoperative hemorrhage during dental procedures [7, 8]. Nevertheless, the literature on dental management of pediatric patients with Type 3 VWD, particularly those requiring comprehensive dental rehabilitation, remains limited. This case report describes the successful management of extensive dental treatment under general anesthesia (GA) in a child with Type 3 VWD, highlighting key aspects of hemostatic planning and clinical execution.

## 2. Case Presentation

### 2.1. Patient Information

A 4‐year‐old male presented to the Pediatric Dentistry Department of Isfahan University of Medical Sciences with a chief complaint of intermittent pain localized to the maxillary left lateral primary incisor. His medical history was significant for a confirmed diagnosis of Type 3 VWD, established through hematologic evaluation. A positive family history was reported, as the patient′s brother was also affected by the same condition and had a history of mucosal bleeding episodes.

### 2.2. Clinical Findings

**Figure 1:**
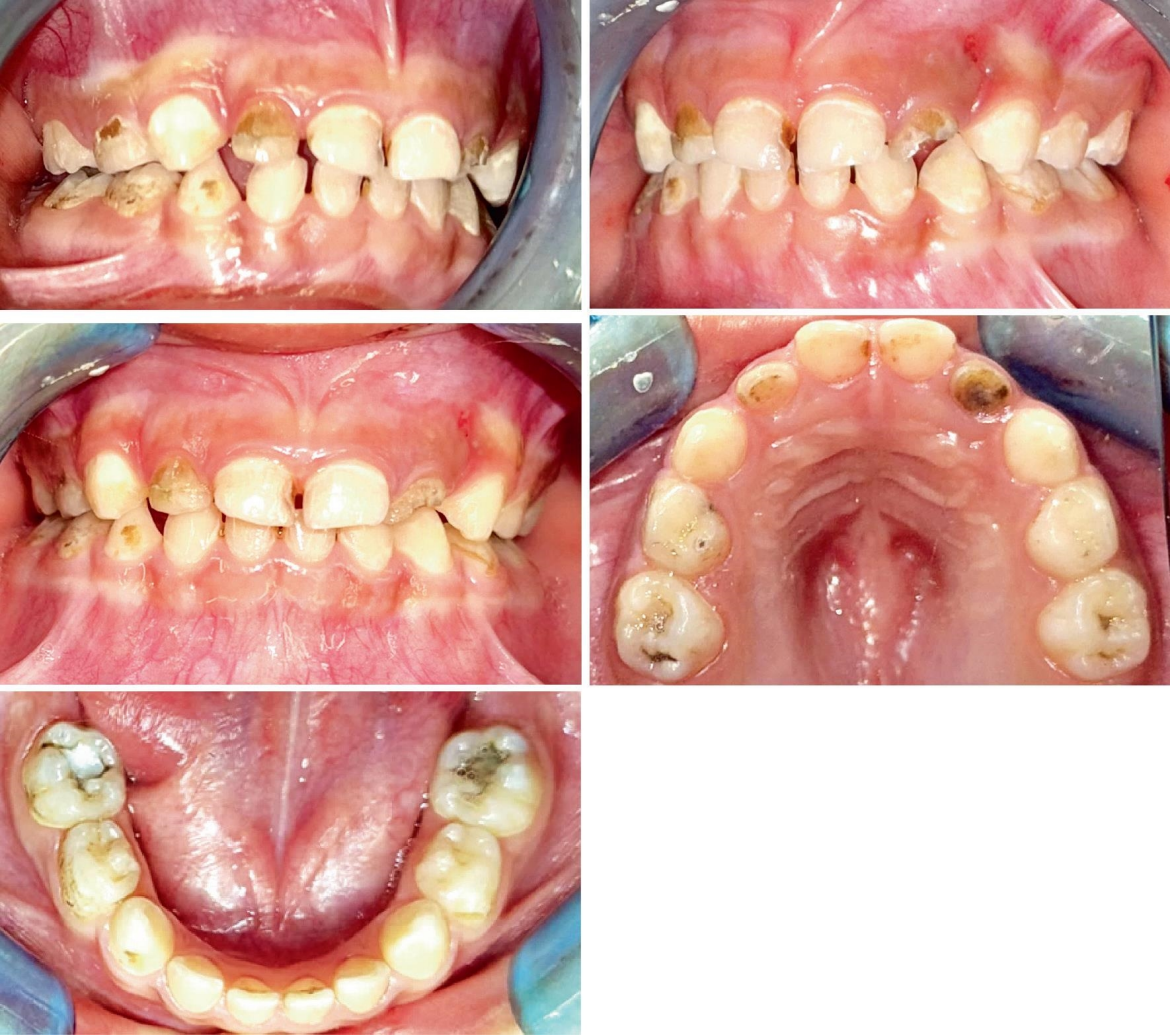
Preoperative intraoral photographs.

Intraoral examination revealed a necrotic maxillary left primary lateral incisor, with no signs of abscess, swelling, or fistula formation. Multiple carious lesions were observed in the remaining primary teeth, caused by poor oral hygiene. The patient exhibited a mesial step molar relationship and a Class I canine relationship. Additionally, two small ecchymotic lesions were noted on the hard palate. Figure 1 illustrates the preoperative intraoral clinical findings. Extraoral examination was unremarkable, and no spontaneous bruising or bleeding was observed on the limbs or joints.

### 2.3. Paraclinical Evaluation

**Figure 2:**
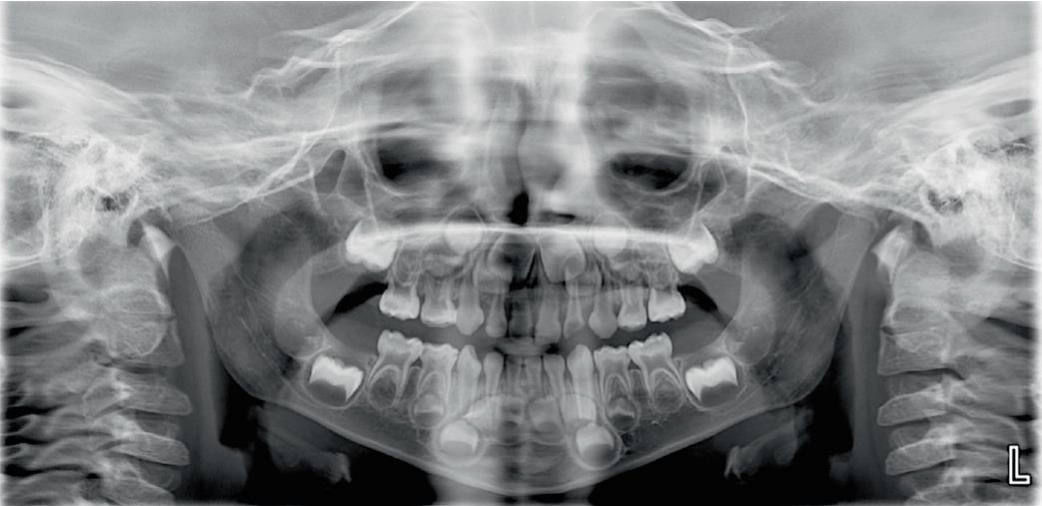
Initial radiograph showing multiple carious lesions in primary teeth.

As shown in Figure 2, a panoramic radiograph demonstrated multiple carious lesions involving Teeth #55, #54, #52, #51, #61, #62, #65, #75, #74, #71, #81, #84, and #85.

**Figure 3:**
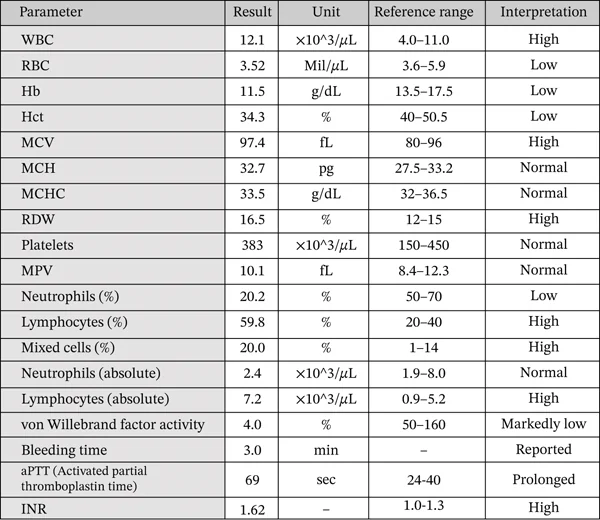
Preoperative laboratory findings in a 4‐year‐old boy with Type 3 von Willebrand disease revealed mild anemia, with low RBC, hemoglobin, and hematocrit values. The coagulation profile also showed markedly reduced von Willebrand factor activity, prolonged aPTT, and a mildly elevated level of INR, whereas the platelet count remained within the normal range.

Preoperative laboratory investigations were performed to evaluate the patient′s hematologic status before GA, as presented in Figure 3. The results revealed markedly reduced FVIII levels and prolonged activated partial thromboplastin time (aPTT, reported as PTT in the laboratory report), consistent with severe Type 3 VWD.

### 2.4. Treatment Planning and Hemostatic Strategy

A comprehensive multidisciplinary treatment plan was developed in close collaboration with the patient′s hematologist. According to the hematology consultation, intravenous administration of one vial of plasma‐derived VWF/F VIII concentrate (Haemate P 1200/500) was recommended before any dental procedure. Repeat dosing every 8–12 h was advised if clinically indicated. For VWD Type 3, Haemate P is administered at a loading dose of 40–80 IU VWF:RCo/kg intravenously, followed by maintenance doses of 30–40 IU VWF:RCo/kg for minor surgical procedures, including dental procedures. Administration is recommended 1–2 h before the procedure and can be repeated every 8–12 h for 2–4 days after oral/minor surgical procedures. The rationale is that Type 3 VWD patients have virtually complete VWF deficiency (levels < 10 IU/dL), making DDAVP ineffective because it releases stored VWF, which is absent. Haemate P contains both VWF and FVIII concentrates in equal proportions, providing the functional VWF needed to increase VWF activity to ≥ 0.50 IU/mL, correct bleeding tendency for platelet adhesion, elevate FVIII levels, and achieve excellent/good clinical response in 97% of surgical patients [9]. GA was considered safe for the patient.

### 2.5. Preoperative Hemostatic Preparation

The patient (18 kg) received one vial of plasma‐derived VWF/FVIII concentrate (Haemate P 1200 IU VWF/500 IU FVIII), corresponding to approximately 67 IU/kg VWF, administered intravenously approximately 15 min before induction of GA to achieve adequate circulating levels of clotting factors.

### 2.6. Dental Management

Full‐mouth dental rehabilitation was performed under GA. Local anesthetic nerve block injections were avoided to minimize the risk of deep soft tissue hematoma; however, buccal infiltration anesthesia was administered using 1.8 mL 2% lidocaine with 1:100,000 epinephrine to assist with intraoperative bleeding control. A rubber dam was placed to protect soft tissues from accidental injury caused by rotary instruments. Pulpotomy was performed using MTA (white ProRoot MTA, Dentsply, Tulsa, Oklahoma, United States) on Teeth #55, #75, #84, and #85, as indicated. Stainless‐steel crowns (SSCs) (MIB, Korea) were placed on all pulpotomized primary molars as well as other carious primary molars. Tooth #64, although free of active caries, exhibited a deep occlusal pit and was prophylactically restored with an SSC. Teeth #52, #51, and #62 underwent pulpectomy using zinc oxide‐eugenol (ZOE), followed by coronal restoration with A1 composite resin (Tokuyama, Japan). Although the panoramic radiograph initially suggested severe root resorption of Tooth #51 and indicated extraction, a preoperative periapical radiograph revealed intact tooth structure. Consequently, extraction was avoided, and pulpectomy was performed to preserve the primary tooth. Tooth #61, which presented with limited carious lesions on its mesial surface, was restored using the same composite material. It is noteworthy that throughout the procedure, meticulous and atraumatic techniques were employed to minimize soft‐tissue injury.

### 2.7. Intraoperative Hemostasis Measures

Gentle pressure and topical antifibrinolytic agents were applied as needed to control localized bleeding. Tranexamic acid was used as a topical antifibrinolytic agent. Tranexamic acid was used at a concentration of 15–25 mg/kg as a mouthwash. This was prepared either from a 10% solution (100 mg/mL) or by crushing 250–500 mg tablets and dissolving them in an appropriate volume of liquid. Application methods generally include mouthwash (rinse for 2 min, 4 times daily, do not swallow), topical soaked gauze held on the bleeding site, direct application of TXA powder (250–500) to the surgical site, or a combination with local hemostats like sutures and gelatin sponges. Antifibrinolytic mouthwash reduces postoperative bleeding significantly without systemic effects [10].

### 2.8. Postoperative Management

The patient was monitored in a controlled recovery setting. No postoperative antifibrinolytic therapy was prescribed after discharge. On the first postoperative night, mild gingival bleeding was observed. In accordance with the hematology consultation, half a vial of plasma‐derived VWF/FVIII concentrate (Haemate P 1200/500), corresponding to approximately 30 IU/kg VWF was administered, which successfully controlled the bleeding. To prevent exacerbation of bleeding, nonsteroidal anti‐inflammatory drugs (NSAIDs) were avoided for postoperative pain management.

### 2.9. Outcomes and Follow‐Up

**Figure 4:**
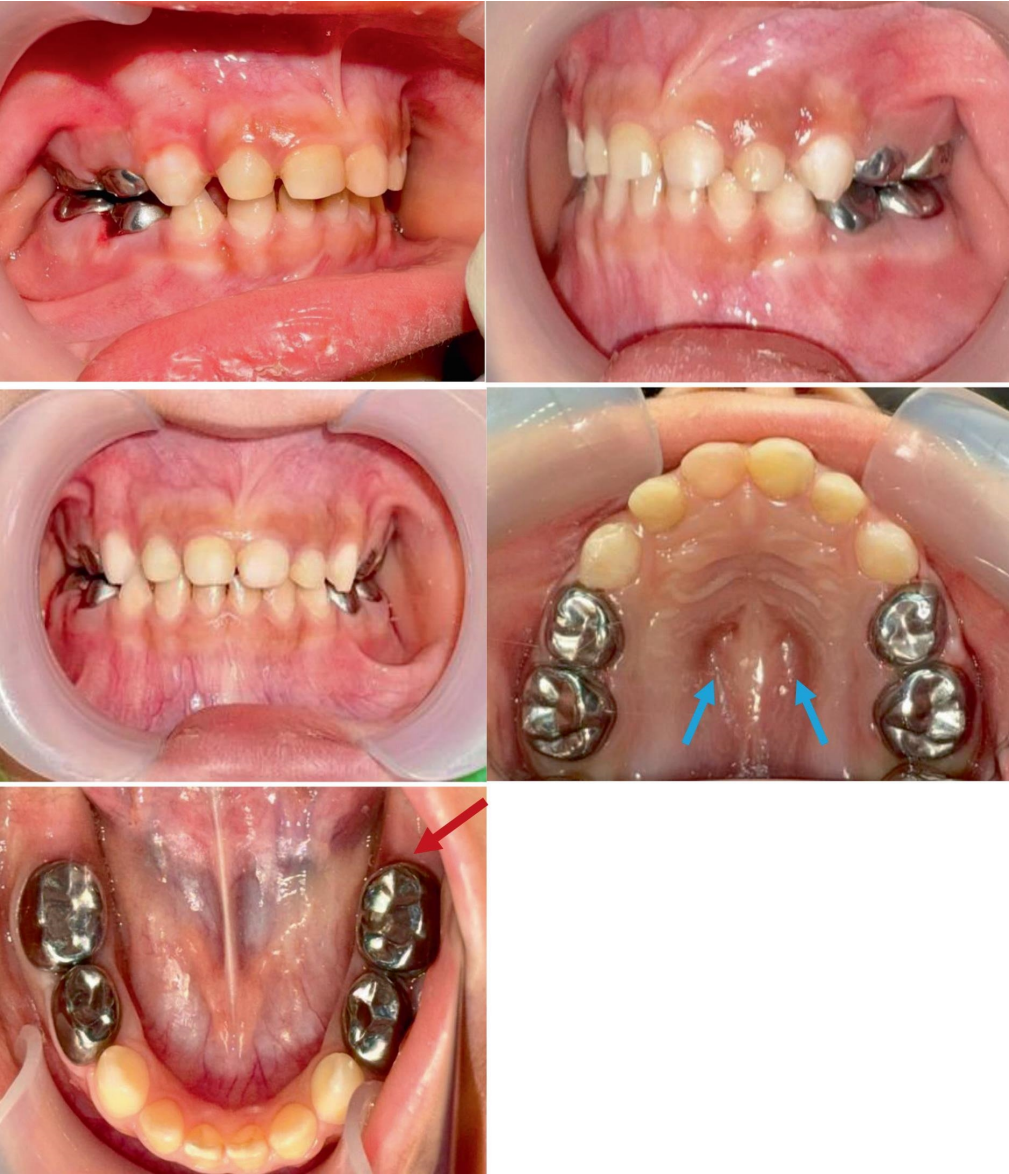
Postoperative intraoral photographs after full‐mouth dental rehabilitation and restored teeth (red arrow). Two small ecchymotic lesions on the hard palate are visible (blue arrow). Atraumatic techniques were employed to minimize soft‐tissue injury.

**Figure 5:**
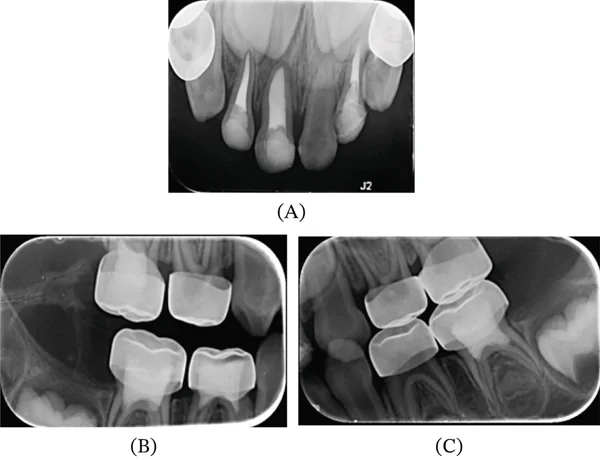
1‐year follow‐up radiographs showing restored primary teeth: (A) Pulpectomy with zinc oxide‐eugenol (ZOE) and composite restoration of Teeth #52, #51, and #62. Tooth #61 was restored with composite resin. (B,C). Pulpotomy with MTA and stainless‐steel crowns (SSC) placement on Teeth #55, #75, #84, and #85. Teeth #54, #64, #65, and #74 were restored with SSC.

The patient recovered uneventfully without major hemorrhagic complications. Comprehensive dental rehabilitation resulted in satisfactory oral function and complete resolution of dental symptoms. Follow‐up examinations at 1 week, 3 months, 6 months, and 12 months revealed intact restorations, stable pulpal outcomes, and sound soft tissues without remarkable complications. Figures 4 and 5 represent the clinical intraoral examination and 1‐year radiographic follow‐up, respectively.

A timeline summarizing the patient′s clinical course, treatment, and follow‐up is presented in Table 1.

**Table 1:** Timeline of clinical events.

Time	Clinical event
Initial visit	Clinical and radiographic examination
Preoperative assessment	Hematology consultation and treatment planning
Day of treatment	Administration of Haemate P and full‐mouth rehabilitation under general anesthesia
Postoperative night	Gingival bleeding occurred and an additional dose of factor concentrate was administered
1‐week follow‐up	Uneventful healing
3 months follow‐up	Clinical stability of restorations
6 months follow‐up	Clinical stability of restorations
12 months follow‐up	Clinical and radiographic stability of restorations

## 3. Discussion

VWD is the most common inherited bleeding disorder worldwide, resulting from quantitative or qualitative defects in VWF. Type 3 VWD represents the most severe form of the disease and is characterized by a marked reduction or complete absence of VWF, leading to elevated aPTT. Although VWD is typically associated with normal prothrombin time (PT) and international normalized ratio (INR), a mild elevation in INR was observed in the present case, which was interpreted cautiously in consultation with the hematology team.

Patients with Type 3 VWD face profound bleeding risks during dental interventions due to severely impaired hemostasis. Multidisciplinary coordination, involving hematologists, anesthesiologists, and dental surgeons, is essential for optimizing outcomes in high‐risk patients with bleeding disorders [11]. Successful management mainly relies on well‐planned replacement therapy, including administration of VWF/FVIII concentrates before and after invasive dental procedures. Deficient VWF can be replaced either alone or in combination with FVIII. VWF/FVIII concentrates are particularly beneficial in patients with low FVIII levels; however, repeated dosing may result in excessively elevated FVIII levels, potentially increasing the risk of thrombosis in individual VWD patients [12]. The timing of factor administration is also critical, as peak levels typically occur approximately 15 min after infusion, providing an optimal window for safe surgical intervention [6, 13].

In this case, adherence to established perioperative protocols, including preoperative plasma‐derived FVIII/VWF (Haemate P) administration and postoperative monitoring, allowed safe pulp therapy and SSC placement under general anesthesia and effectively prevented significant postoperative bleeding. Reports have suggested successful invasive treatments, including tooth extraction with recombinant VWF replacement, without postoperative bleeding in Type 3 VWD patients [3, 14]. In contrast, Dudek et al. [15] reported a successful apicoectomy procedure in a patient with Type 1 VWD without perioperative hemostatic replacement therapy. Unlike the mild bleeding phenotype of Type 1 VWD, our patient had Type 3 VWD, the most severe form of the disease, which requires more intensive hemostatic management. These findings highlight that dental procedures can be safely accomplished across different severities of VWD when individualized treatment protocols and close collaboration with hematologists are implemented.

Additionally, topical antifibrinolytic agents, such as tranexamic acid, are provided in high local concentrations that inhibit fibrinolysis and further support intraoperative hemostasis [8]. These agents, along with meticulous local hemostatic measures, further reduce intraoperative bleeding and can decrease the need for additional factor replacement when used in combination with systemic preparation [16].

Dental care in patients with bleeding disorders is challenging because even minor procedures can provoke prolonged bleeding. Although routine restorative procedures are generally considered low risk, precautions should be taken to minimize trauma to gingival tissues during the placement of rubber dam clamps, matrices, and wedges. Additionally, the use of a rubber dam is recommended to protect soft tissues from accidental injury caused by rotatory or sharp instruments. Whenever feasible, endodontic treatment is preferred over extraction, as it generally carries a lower bleeding risk and can be performed using standard clinical protocols [17]. In the present case, Tooth #62, although necrotic, was preserved via pulpectomy rather than extraction because the root structure was intact, and maintaining the primary tooth minimized the risk of surgical bleeding. Al‐Zubaidi [18] has reported an effective endodontic treatment as a tooth‐preserving approach in a patient with VWD. Furthermore, the posterior teeth, including some with minimal caries and even a clinically intact tooth (#64), were restored with SSCs using a relatively aggressive preventive approach. This strategy was guided by the patient′s young age, underlying hematologic condition, and the need to provide definitive treatment under general anesthesia to prevent future interventions. Consistent with our experience, a retrospective cohort of children with Type 3 VWD demonstrated a high frequency of dental disease and demonstrated the necessity of factor replacement in diverse dental procedures [19].

Reported rates of postoperative bleeding after invasive dental procedures range from 0.2% to 3.3% in patients with normal hemostasis and from 8.6% to 32.1% in those with bleeding disorders [20]. A large cohort study of 100 VWD patients treated with Haemate P reported 97% effective hemostatic outcomes in surgical and dental procedures [21]. In a retrospective study, Hazendonk et al. [22] concluded that Haemate P is effective in preventing bleeding during invasive procedures. In the present case, an additional postoperative dose of factor concentrate was required, which may be attributed to the pharmacokinetic half‐life of the administered VWF/FVIII concentrate. Similarly, retrospective data indicate that a tailored factor replacement regimen for dental procedures was required, underscoring the importance of individualized dosing based on the extent of the procedure and pharmacokinetic considerations [8].

It should be noted that the use of acetylsalicylic acid and NSAIDs is generally discouraged in patients with bleeding disorders, including VWD, due to their effects on platelet function; however, this restriction primarily concerns prolonged use [23].

This case report contributes to the growing body of evidence that comprehensive dental rehabilitation under general anesthesia can be safely performed in pediatric patients with Type 3 VWD when careful hemostatic planning, multidisciplinary coordination, and thoughtful treatment strategies are employed. Importantly, individualized approaches, such as preserving necrotic teeth via pulpectomy and employing preventive restorative measures on posterior teeth, can minimize surgical trauma and the risk of future interventions, respectively.

## 4. Conclusion

Dental care for patients with severe inherited bleeding disorders, such as Type 3 VWD, requires proactive hematologic and meticulous perioperative factor management. This case demonstrates that comprehensive dental treatment under general anesthesia, combined with appropriate factor replacement and hemostatic strategies, can be performed safely in a 4‐year‐old child with Type 3 VWD.

## Author Contributions

Samira Sadigh Batha: Conceptualization, methodology, investigation, data curation, and writing—review and editing. Nazanin Nasr: Conceptualization, investigation, data curation, and writing—eriginal draft. Nasser Kaviani: Supervision, validation, and writing—review and editing.

## Funding

No funding was received for this manuscript.

## Disclosure

All authors have read and approved the final version of the manuscript. Nazanin Nasr had full access to all of the data in this study and takes complete responsibility for the integrity of the data and the accuracy of the data analysis.

## Consent

According to institutional regulations, ethical approval was not required for this single anonymized case report. Written informed consent for publication of the patient′s clinical information and accompanying images was obtained from the patient′s parent/legal guardian.

## Conflicts of Interest

The authors declare no conflicts of interest.

## Supporting information


**Supporting Information** Additional supporting information can be found online in the Supporting Information section. File S1: Checklist of information to include when writing a case report.

## Data Availability

The data that support the findings of this study are available from the corresponding author upon reasonable request.
