# Supplementary material for: Comprehensive Dental Management Under General Anesthesia in a Pediatric Patient With Type 3 von Willebrand Disease: A Case Report
Source: Case Rep Dent. 2026 Sep 7;2026:4211452. doi: 10.1155/crid/4211452 (PMC13548132; doi:10.1155/crid/4211452)
Supplement: Supplementary file 1 [file CRID-2026-4211452-s001.pdf]

CARE case report guidelines

CARE Checklist of information to include when writing a case report

(cc) BY-NC-ND

Topic	Item	Checklist item description	Reported on Line
Title	1	The diagnosis or intervention of primary focus followed by the words "case report"	page1, line 1
Key Words	2	2 to 5 key words that identify diagnoses or interventions in this case report, including "case report"	page1, line 18-19
Abstract (no references)	3a	Introduction: What is unique about this case and what does it add to the scientific literature?	page1, line 4-7
	3b	Main symptoms and/or important clinical findings	page1, line 8-10
	3c	The main diagnoses, therapeutic interventions, and outcomes	page1, line 10-14
	3d	Conclusion—What is the main "take-away" lesson(s) from this case?	page1, line 15-17
Introduction	4	One or two paragraphs summarizing why this case is unique (**may include references**)	page1, line 31-35
Patient Information	5a	De-identified patient specific information.	page3, line 2-4
	5b	Primary concerns and symptoms of the patient.	page3, line 4
	5c	Medical, family, and psycho-social history including relevant genetic information	page3, line 4-8
	5d	Relevant past interventions with outcomes	N/A
Clinical Findings	6	Describe significant physical examination (PE) and important clinical findings.	page3, line 9-15
Timeline	7	Historical and current information from this episode of care organized as a timeline	table 1
Diagnostic Assessment	8a	Diagnostic testing (such as PE, laboratory testing, imaging, surveys).	figures 2 and 3
	8b	Diagnostic challenges (such as access to testing, financial, or cultural)	N/A
	8c	Diagnosis (including other diagnoses considered)	N/A
	8d	Prognosis (such as staging in oncology) where applicable	N/A
Therapeutic Intervention	9a	Types of therapeutic intervention (such as pharmacologic, surgical, preventive, self-care)	page5, line 1-6
	9b	Administration of therapeutic intervention (such as dosage, strength, duration)	page1, line 7-9
	9c	Changes in therapeutic intervention (with rationale)	page 5, line 12, 14, 24-25, 29-30
Follow-up and Outcomes	10a	Clinician and patient-assessed outcomes (if available)	page5, line 38-41
	10b	Important follow-up diagnostic and other test results	figures 4 and 5
	10c	Intervention adherence and tolerability (How was this assessed?)	N/A
	10d	Adverse and unanticipated events	N/A
Discussion	11a	A scientific discussion of the strengths AND limitations associated with this case report	page9, line 11-16
	11b	Discussion of the relevant medical literature **with references**.	page8, line 39-42. page9 line 1-2
	11c	The scientific rationale for any conclusions (including assessment of possible causes)	page 9, line3-4
	11d	The primary "take-away" lessons of this case report (without references) in a one paragraph conclusion	page9, line 17-21
Patient Perspective	12	The patient should share their perspective in one to two paragraphs on the treatment(s) they received	N/A
Informed Consent	13	Did the patient give informed consent? Please provide if requested	Yes ☒ ** No ☐
